# SNHG14 induces osteogenic differentiation of human stromal (mesenchymal) stem cells in vitro by downregulating miR-2861

**DOI:** 10.1186/s12891-020-03506-9

**Published:** 2020-08-08

**Authors:** Mingchang Du, Bo Wu, Shiwen Fan, Ye Liu, Xu Ma, Xun Fu

**Affiliations:** The Orthopedic Hospital of Shenyang, No. 115 Dong bei da ma lu road, Da dong district of Shenyang, Shenyang City, Liaoning Province 110000 PR China

**Keywords:** SNHG14, Osteogenic differentiation, Human stromal (mesenchymal) stem cells, miR-2861

## Abstract

**Background:**

The differentiation of human stromal (mesenchymal) stem cells (hMSCs) is a critical procedure for the development of osteoblast. SNHG14 is a newly discovered lncRNA that has been barely studied. Our preliminary experiments showed that SNHG14 may be dysregulated in the differentiation of hMSCs. In this study, we focused on elucidating the relationships among SNGH14, miR-2861, and osteoblastic differentiation of hMSCs.

**Method:**

To investigate the roles of SNHG14 and miR2861 in hMSCs differentiation, qRT-PCR, luciferase activity, cell transfections, the detections of ALP activity, and Alizarin Red staining were performed.

**Result:**

We found that the expression of SNHG14 was enhanced, while the expression of miR-2861 was suppressed in serum and hMSCs from patients with osteoporosis. SNHG14 could target miR-2861, and shSNHG14 suppressed osteoblast differentiation of hMSC. MiR-2861 suppressed osteoblast differentiation of hMSC. In addition, the effects of SNHG14 on osteoblast differentiation of hMSC were attenuated by miR-2861.

**Conclusion:**

In conclusion, our experimental data showed that the induction effects of SNHG14 on osteoblast differentiation of hMSC were attenuated by miR-2861. SNHG14 could induce osteogenic differentiation of hMSC in vitro by targeting miR-2861.

## Background

Mesenchymal stem cells have the capabilities of self-renewal and multi-lineage differentiation, which are critical factors in the regeneration or repairment of bone tissues [1, 2]. Human bone marrow mesenchymal stem cell (hMSCs) could fully differentiate to many cell types including osteoblasts, chondrocytes, and adipocytes [3, 4]. The differentiation of hMSCs is thus critical for the development of osteoblast. Studies have modulated the cell signaling pathways to control the differentiation of hMSCs to osteoblasts [5, 6]. However, the underlying mechanisms remain to be elusive.

Non-coding RNAs have become the hotspot in several research fields, including long non-coding RNAs (lncRNAs) (> 200 nt) [7] and microRNAs (miRNAs) (~ 20 nt) [8]. Various lncRNAs have been reported to be involved in the osteoblastic differentiation of hMSCs. For instance, down-regulation of lncRNA-ANCR promoted osteoblast differentiation by targeting EZH2 and regulating the expression of Runx2 [9]. LncRNA H19 was reported to mediate BMP9-induced osteogenic differentiation of MSCs through the Notch signaling [10]. LncRNA SNHG14 is a newly discovered lncRNA that has been barely demonstrated regarding its biological roles in human diseases. It was reported that SNHG14 promoted microglia activation by regulating miR-145-5p/PLA2G4A in cerebral infarction [11]. Very limited information has been revealed for its functions in hMSCs.

MiRNAs are another group of non-coding RNAs that have been widely reported in human diseases. Many miRNAs exert essential roles in the differentiation of hMSCs to osteoblast. For example, microRNA-138 was revealed to regulate the osteogenic differentiation of human stromal (mesenchymal) stem cells in vivo [6]. Another study also reported that the microRNA-320/RUNX2 axis regulates adipocytic differentiation of human mesenchymal (skeletal) stem cells [12]. Moreover, miR2861 has been demonstrated to participate in the regulatory feedback loop during differentiation of mouse osteoblast [13].

From our preliminary experiment, we noticed that SNHG14 may be dysregulated in hMSCs differentiation, and miR2861 may share the common binding sequences with lncRNA SNHG14. In this study, we aimed to clarify the role of lncRNA SNHG14 in the formation of osteoblast from hMSCs focusing on elucidating the relationships among SNGH14, miR2861, and osteoblastic differentiation of hMSCs.

## Methods

### Human samples

In this study, patients with hip fracture were recruited at The Orthopedic Hospital of Shenyang. Patient samples were divided into two groups (6 patients in each group), including the treatment group (osteoporosis patients with a fracture) and the control group (non-osteoporosis patients with a fracture). Serum and bone tissues were collected during endoprosthesis, and gamma nail was implanted into the proximal femur. All patients enrolled in this study signed the informed consent. This study was approved by the Research Ethics Committee of The Orthopedic Hospital of Shenyang.

### hMSC preparations

hMSCs were obtained from the bone marrow from femurs of 4 patients during total hip or knee arthroplasty due to osteoarthritis or hip fracture. The Ethics Review Board of Orthopedic Hospital of Shenyang, Shenyang City, Liaoning Province approved our study. All hMSCs were obtained from postmenopausal women with an average age of 68.5 years old (age range 60–77 years old). Densitometric examinations were performed using a Lunar iDXA apparatus (GE Lunar, Madison, WI, USA). Diagnosis of osteopenia or osteoporosis were made using the WHO T-score criteria (− 2.5 < T-score < − 1 or T-score ≤ − 2.5, respectively). All the subjects in the osteoporosis group had vertebral fractures.

### Cell separation

The RosetteSep Isolation kit (STEMCELL, Canada) was used to isolate hMSCs. Cells were cultured at 37 °C in a wet environment with 5% CO_2_. The culture medium was refreshed every week. When cells reached confluence, they were trypsinized and used immediately.

### Cell culture

We cultured hMSCs in α-minimum essential medium (αMEM) containing 10% fetal bovine serum (FBS Invitrogen), antibiotics, and glutamax I (GIBCO, USA). Osteogenesis was induced by fresh osteoblast induction medium (OIM) with 10^− 8^ M dexamethasone (Sigma-Aldrich, D4902), 0.2 mM l-ascorbic acid (Sigma-Aldrich, A8960), 10 mM β-glycerophosphate (Sigma-Aldrich, G9422), and 10 mM 1.25-vitamin-D3. Alkaline phosphatase (ALP) was used to assess osteoblast phenotype. Alizarin Red staining was used to test matrix mineralization. The medium was changed every 3 d throughout the experiments and cells were harvested at indicated time points.

### qRT-PCR

Total RNAs were extracted from serum, bone tissues or hMSCs by Trizol (Invitrogen, USA). The Reverse Transcription Kit (Applied Bio., USA) was used to synthesize cDNAs. The qRT-PCR reactions were prepared using SYBR Select Master Mix (Applied Bio., USA) and PCR was carried out on an ABI 7900-fast thermocycler (Applied Bio., USA). The relative expression was calculated by 2^-ΔΔCT^ method. The sequences of the primers are listed below.

SNHG14-F: 5′-GGGTGTTTACGTAGACCAGAACC-3′;

SNHG14-R: 5′-CTTCCAAAAGCCTTCTGCCTTAG-3′;

GAPDH-F: 5′-GAAGGTGAAGGTCGGAGTC-3′;

GAPDH-R: 5′-GAAGATGGTGATGGGA TTTC-3′.

OC-F -F 5′-GGCGCTACCTGTATCAATGG-3′;

OC-R 5′-GTGGTCAGCCAACTCGTCA-3′.

Runx2-F: 5′-CGAATAACAGCACGCTATTAA-3′.

Runx2-R: 5′-GTCGCCAAACAGATTCATCCA-3′.

OSX-F: 5′-GCCAGAAGCTGTGAAACCTC-3′;

OSX-R: 5′-GCTGCAAGCTCTCCATAACC-3′;

ALP-F: 5′-TAGTGAAGAGACCCAGGCGCT-3′;

ALP-R: 5′-ATAGGCCTCCTGAAAGCCGA-3′;

miR-2861-F: 5′-AACGAGACGACGACAGAC-3′;

miR-2861-R: 5′-GGGGCCUGGCGGUGGGCGG-3′;

U6: 5′-GCCCCCGCCTCCGCCGCCGCC-3′ and 5′-ATATGGAACGCTTCACGAATT-3′.

### Cell transfections

Vectors with sh-SNHG14, miR-2861 mimic, and miR-2861 inhibitor (all from Genepharma) were transfected to hMSCs via Lipofectamine 2000 (Sigma, USA). At 2 d post-transfection, qRT-PCR was conducted to detect gene expressions. The miR-2861 mimic sequence was 5′-GGGGCCUGGCGGCGGGCGG-3′. Mimic control sequence was 5′-UUCUCCGAACGUGUCACGUTT-3′. The antagomir sequence was 5′-CCGCCCGCCGCCAGGCCCC-3′. The antagomir control sequence was 5′-CAGUACUUUUGUGUAGUACAA-3′.

### ALP activity

hMSCs were collected and washed. The cells were lysed by 1% Triton X-100 for 15 min and centrifuged at 10,000 g for 5 min. The supernatant was used for ALP analysis by ALP Assay Kit (Abcam, USA).

### Alizarin red staining

The osteoblasts were cultured by OIM for 2 weeks and then fixed by 70% ethanol. Next, the cells were incubated by 0.5% Alizarin Red solution for an hour at 25 C. The results were recorded for analysis.

### Luciferase assay

Primers were designed for the potential miR-2861 binding sequence of AKT2 3′-UTR, SNHG14 3′-UTR, and then cloned into the Sac I/Xba I sites of pmirGLODual-Luciferase reporter vector. The reconstructed plasmids were confirmed by sequencing and named pmirGLO/SNHG14-WT and pmirGLO/AKT2-wt1. We also commercially synthesized mutant reporter constructs by mutating three nucleotides of each potential miR-2861 binding site and designated as pmirGLO/SNHG14-MUT, pmirGLO/AKT2-mut1. Cells of 90% confluence were seeded in triplicate in 96-well plates. The wild-type (WT) or mutant reporter constructs (Mut) were co-transfected into SiHa cells in the 96-well plates with 50 nmol/L miR-2861 or 50 nmol/L miR-NC by using lipofectamine 2000 (Invitrogen, CA, USA), respectively. Reporter gene assays were performed 24 h post-transfection using the Dual-Luciferase Reporter Assay Kit (Promega) following the manufacturer’s instructions. Firefly luciferase activity values were normalized for transfection efficiency using the corresponding Renilla luciferase activity. Three independent experiments were performed.

### Western blot analysis

Cell protein lysates were separated in 8% or 10% SDS-PAGE gel 72 h post-transfection, followed by transferring to polyvinylidene difluoride membrane (PVDF). Western blot analysis was performed with monoclonal anti-p53 (Santa Cruz), anti-AKT2 (Abcam) primary antibodies. Anti-GAPDH antibody (Santa Cruz) was used as an internal control. The membrane was washed and incubated with horseradish peroxidase (HRP)-conjugated secondary antibody (Cell Signaling Technology, USA). Complexes were visualized with SuperSignal West Pico Chemiluminescent Substrate (Pierce) and the expression levels of these proteins were evaluated by Quantity One software.

### Statistical analysis

Data were shown as mean ± stand deviation (SD). Comparisons were performed by *t*-test (between 2 groups) or one-way ANOVA (among multiple groups). *P* < 0.05 was considered statistical significant differences.

## Results

### SNHG14 was upregulated but miR-2861 was downregulated in serum and hMSCs from patients with osteoporosis

**Fig. 1:**
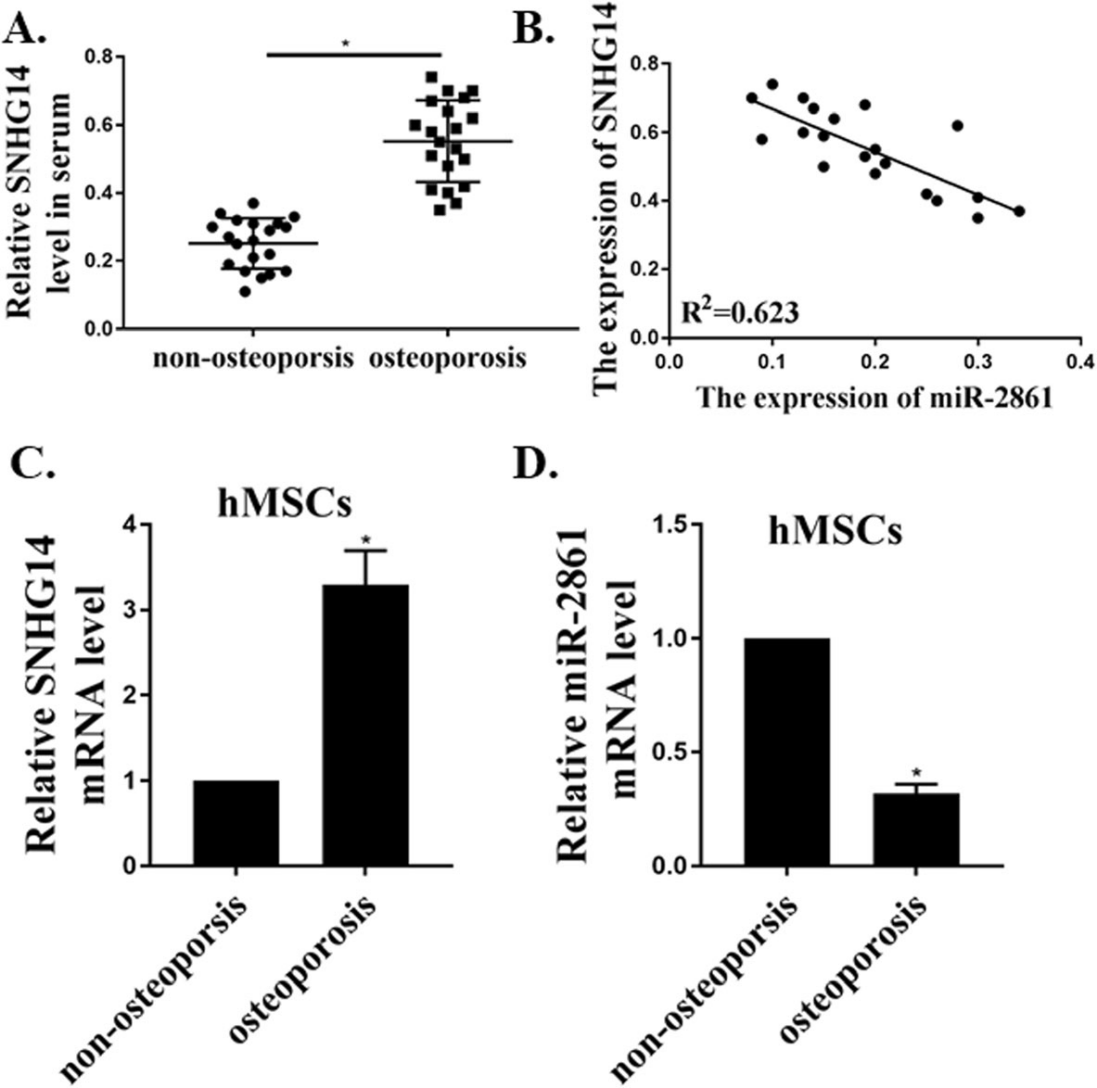
SNHG14 was upregulated but miR-2861 was downregulated in serum and hMSCs from patients with osteoporosis. **a**. Expressions of SNHG14 in the serum of non-osteoporosis people and osteoporosis patients (*n* = 20). **b**. The negative relationship between the expression of SNHG14 and miR-2861 in the serum of osteoporosis patients (n = 20). **c**. Expression of SNHG14 in hMSCs of non-osteoporosis people and osteoporosis patients (*n* = 4). **d**. Expression of miR-2861 in hMSCs of non-osteoporosis people and osteoporosis patients (*n* = 4). * *p* < 0.05

The expression of SNHG14 and miR-2861 in serum and hMSCs of osteoporosis patients were analyzed. Compared to participants without osteoporosis (*n* = 20), the expression levels of SNHG14 in serum and hMSCs of osteoporosis patiens (*n* = 20) were greatly elevated (Fig. 1a and c). In addition, the expression of miR-2861 was dramatically down-regulated in hMSCs of osteoporosis group (Fig. 1d). In addition, a negative relationship between the expression of SNHG14 and miR-2861 in the serum of the osteoporosis group was observed (Fig. 1b).

### SNHG14 was targeted by miR-2861

**Fig. 2:**
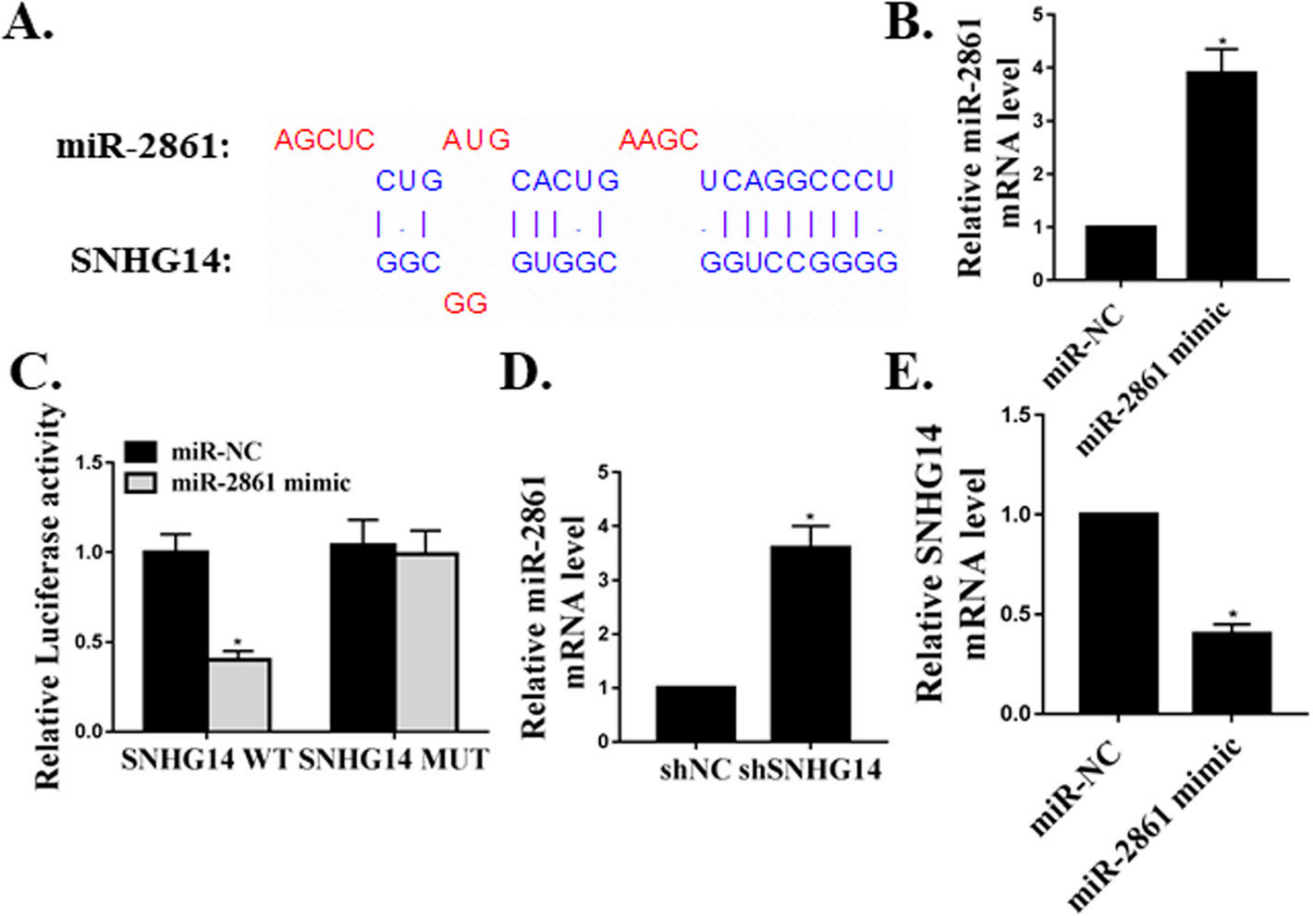
SNHG14 was targeted by miR-2861. **a**. Common binding sequences between SNHG14 and miR-2861. **b**. Expression of miR-2861 mRNA in hMSCs. **c**. Dual-luciferase reporter assay. **d**. Expression of miR-2861 mRNA in hMSCs. **e**. Expression of SNHG14 mRNA in hMSCs. *N* = 3, *p < 0.05

We further investigated the relationship between SNHG14 and miR-2861. As shown in Fig. 2a, the common binding site between SNHG14 and miR-2861 was observed. After successfully transfecting miR-2861 into hMSCs (Fig. 2b), the co-transfection of SNHG14 3′-UTR with miR-2861 led to the suppression of luciferase activities compared with that of SNHG14 MUT (Figue 2C). Moreover, the transfection of shSNHG14 elevated the expression levels of miR-2861 (Fig. 2d). The expression levels of SNHG14 were also reduced in cells transfected with miR-2861 (Fig. 2e). Thees data indicated that SNHG14 was targeted by miR-2861.

### shSNHG14 suppressed osteoblast differentiation of hMSC

**Fig. 3:**
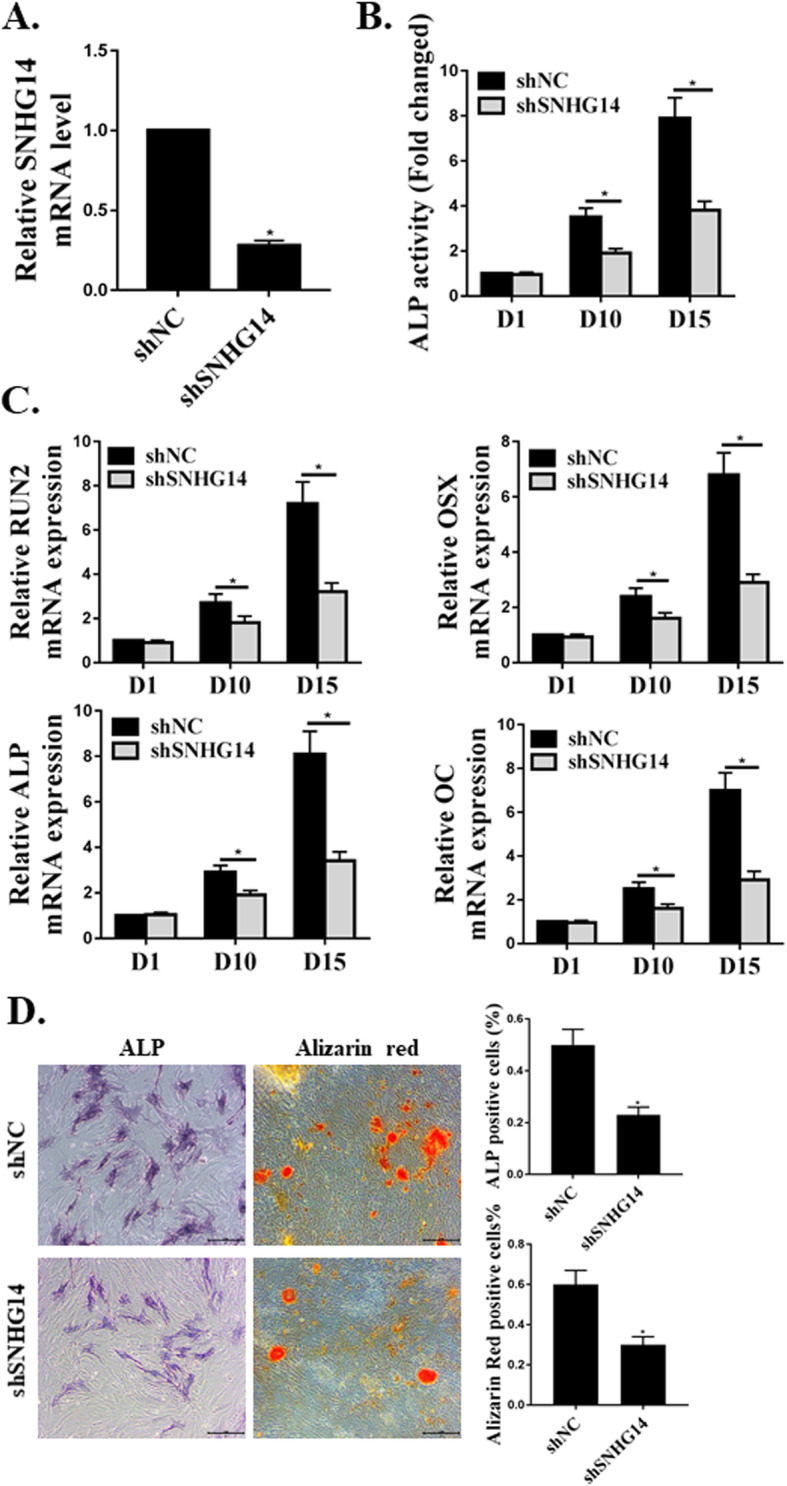
shSNHG14 suppressed osteoblast differentiation of hMSC. **a**. The expression of SNHG14 mRNA in hMSCs. **b**. ALP activities in shSNHG14 or shNC transfected hMSCs on day 1, day 10, and day 15. **c**. Osteoblast differentiation assessed through osteoblast marker genes of *RUNX2*, *OSX*, *ALP*, and *OC* normalized to β-actin on day 1, day 10, and day 15. **d**. ALP and Alizarin Red staining on day 15. *N* = 3, *p < 0.05

To investigate the effects of SNHG14 on hMSC osteoblast differentiation, we induced hMSCs differentiation to osteoblasts after transfection with shSNHG14 or shNC. As shown in Fig. 3a, the expression levels of SNHG14 were reduced in cells transfected with shSNHG14. The suppression of SNHG14 markedly lowered osteoblastic differentiation, which was indicated by lower expression levels of the osteoblast-specific genes *RUNX2*, *Osterix* (*OSX*), *ALP*, *OC*, and decreased ALP activity (Figs. 3b-d). We observed matrix mineralization in vitro by Alizarin red staining in shSNHG14–transfected hMSCs compared with cells transfected with shNC. It was obvious that shSNHG14 could suppress hMSCs differentiation to osteoblasts 2 weeks post-transfection.

### MiR-2861 suppressed osteoblast differentiation of hMSC

**Fig. 4:**
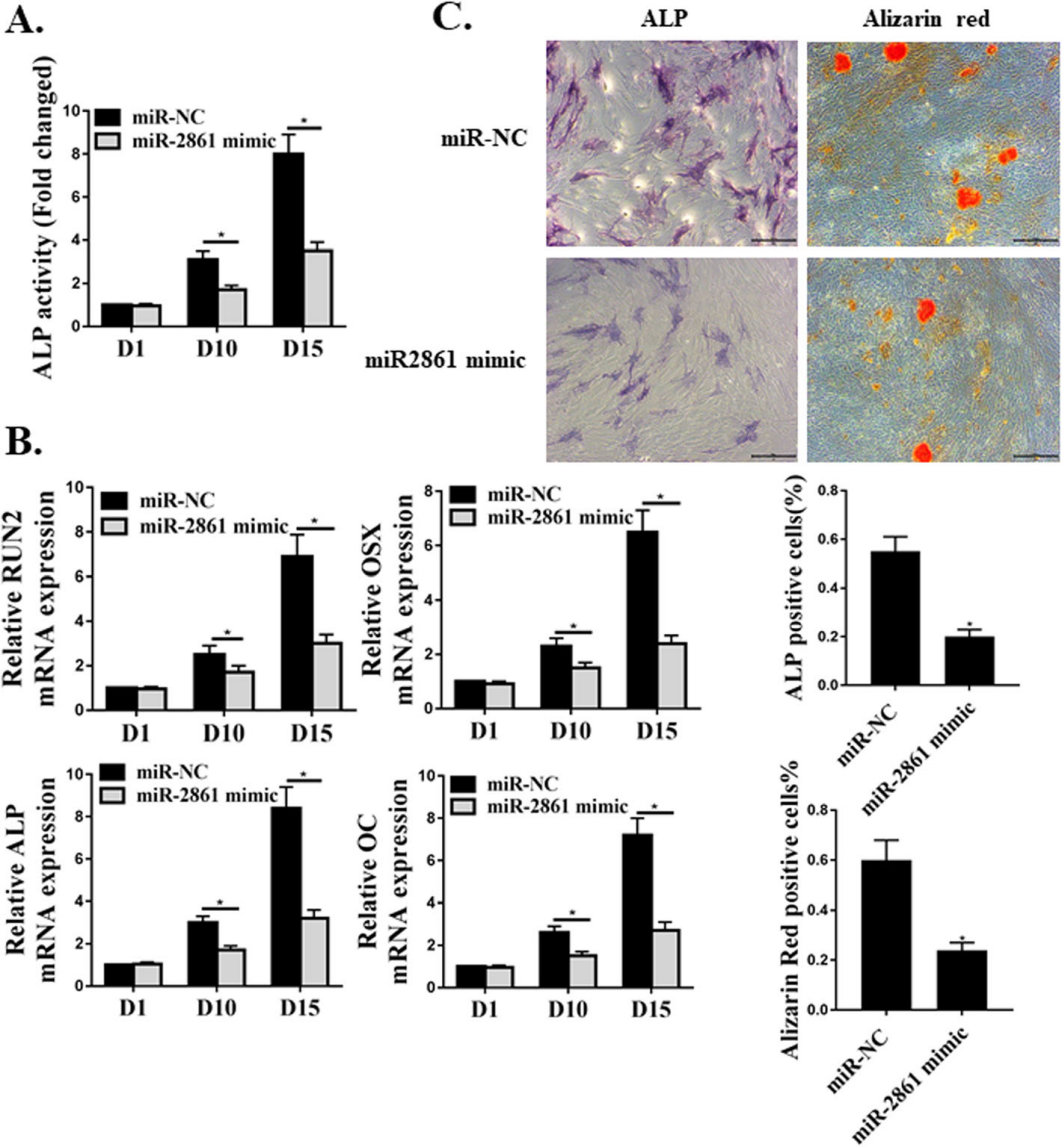
MiR-2861 suppressed osteoblast differentiation of hMSC. **a**. ALP activities measured at day 1, day 10, and day 15 of osteoblast differentiation. **b**. osteoblast differentiation assessed by the mRNA expression of *RUNX2*, *OSX*, *ALP*, and *OC* day 1, day 10, and day 15. **c**. ALP and Alizarin Red staining results on day 15. *N* = 3, *p < 0.05

To further evaluate the effects of miR-2861 on hMSC osteoblast differentiation, we induced hMSCs to differentiate to osteoblasts after transfection with miR-2861-mimic or miR-NC. Over-expression of miR-2861 significantly suppressed osteoblastic differentiation, which was indicated by decreased ALP activity (Fig. 4a), lower expression levels of *RUNX2*, *OSX*, *ALP*, and *OC* (Fig. 4b), and reduced in vitro matrix mineralization (Fig. 4c) in miR-2861-mimic transfected hMSCs, in contrast to cells transfected with miR-NC.

### The effects of SNHG14 on osteoblast differentiation of hMSC were attenuated by miR-2861

**Fig. 5:**
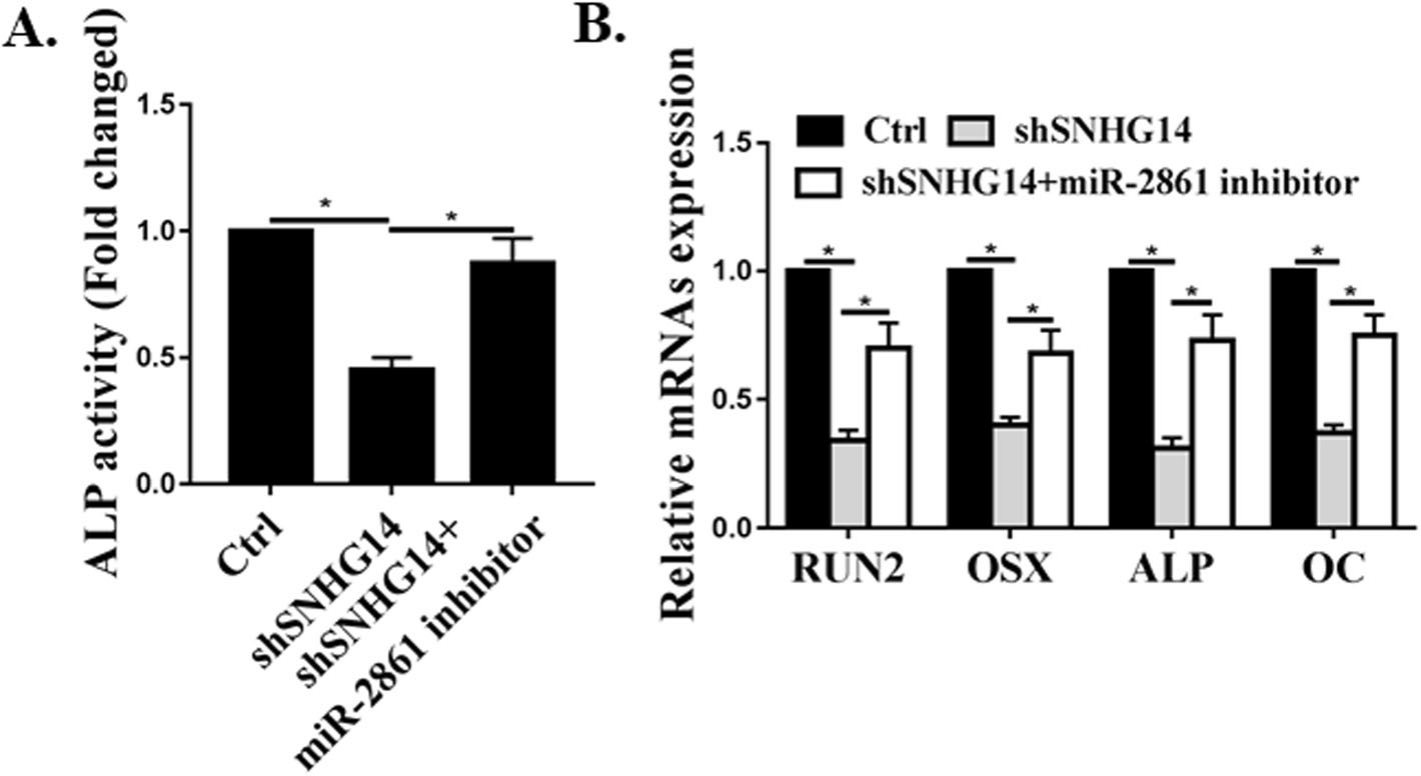
The effects of SNHG14 on osteoblast differentiation of hMSC were attenuated by miR-2861. **a**. ALP activities in cells transfected with control, shSNHG14, or shSNHG14 + miR-2861-inhibitor at day 15. **b**. Expression of osteoblast marker genes of *RUNX2*, *OSX*, *ALP*, and *OC* at day 15. *N* = 3, *p < 0.05

Whether miR-2861 could attenuate the effects of SNHG14 on osteoblast differentiation of hMSC. Figure 5a illustrated that shSNHG14 decreased ALP activity but the effects were attenuated by co-transfection with miR-2861 inhibitor. Figure 5b demonstrated that down-regulation of miR-2861 greatly lowered osteoblastic differentiation induced by shSNHG14, since shSNHG14 decreased osteogenesis.

### AKT2 was targeted by miR-2861

**Fig. 6:**
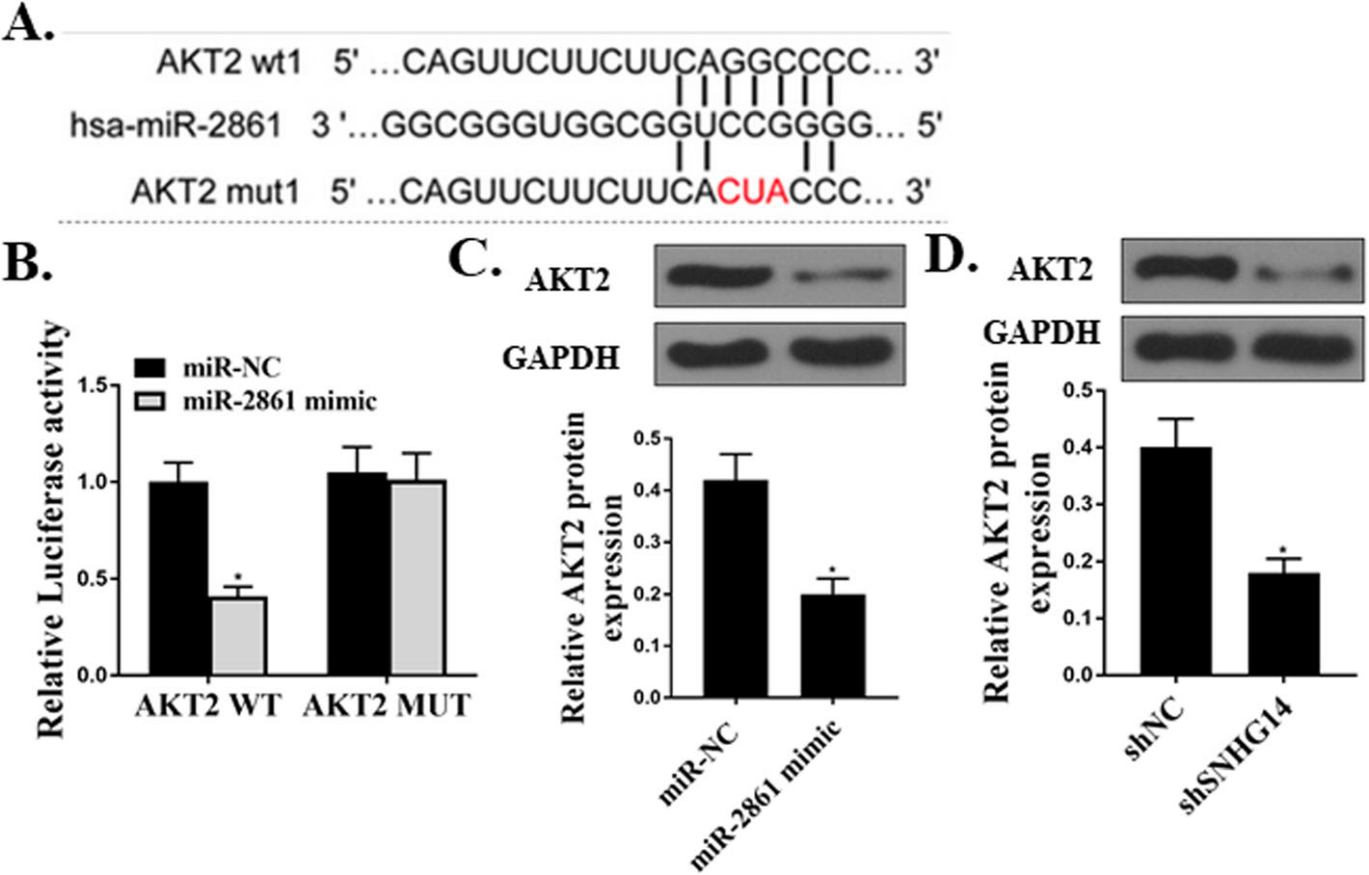
AKT2 was targeted by miR-2861. **a**. Shared binding sequences between AKT2 and miR-2861. **b**. Dual-luciferase reporter assay. **c** and **d**. Western blot assay of AKT2 protein expression levels. *N* = 3, *p < 0.05

Finally, the mechanisms by which miR-2861 functioned to affect the differentiation of hMSCs were explored. Our bioinformatics analysis and luciferase assay results showed that AKT2 could bind with miR-2861 (Fig. 6a and b). In addition, overexpression of miR-2861 decreased the expression levels of AKT2, and down-regulation of SNHG14 reduced the expression of AKT2 (Fig. 6c and d).

## Discussions

Osteoblastic differentiation from hMSCs many originates from many cell events that are affected by various molecular and cellular procedures during the development of bone and skeleton. It is crucial to reveal important factors that mediate this phenomenon, and to study the underlying mechanisms. Owing to the successful findings from the previous studies, different lncRNAs have been shown to participate in the osteoblast differentiation by targeting corresponding cell signaling pathways. One study revealed the expression profiling of lncRNAs in C3H10T1/2 mesenchymal stem cells undergoing early osteoblast differentiation [14]. LncRNA H19 promoted osteoblast differentiation via the TGF-β1/S mad3/HDAC signaling pathway by deriving miR-675 [15].

Various lncRNAs and miRNAs are dysregulated during the hMSCs differentiation of osteoblast [16, 17]. In our study, we found a similar phenomenon. We firstly analyzed the expression of SNHG14 and miR-2861 in serum and hMSCs of osteoporosis patients. Compared to non-osteoporosis participants, the expression levels of SNHG14 in serum and hMSCs of osteoporosis patients were greatly elevated. The expression of miR-2861 was drastically down-regulated in hMSCs of osteoporosis group. A negative relationship was established between the expression of SNHG14 and miR-2861 in serum of osteoporosis group. Similar to previous studies, we identified that lncRNA SNHG14 was upregulated but miR-2861 was downregulated in serum and hMSCs from patients with osteoporosis.

With the common shared binding sequences, lncRNAs could target their specific miRNAs and exert the biological roles in the pathogenesis of many cellular procedures [18]. For example, lncRNA DGCR5 acts as a tumor suppressor in papillary thyroid carcinoma via targeting miR-2861 [17]. We first confirmed the common binding sequences between SNHG14 and miR-2861. Co-transfection of SNHG14 3′-UTR with miR-2861 led to the suppression of luciferase activities compared with that of SNHG14 MUT. Moreover, shSNHG14 elevated the expression levels of miR-2861. The relative expression levels of SNHG14 were also lowered in cells transfected with miR-2861. As far as we know, we are the first to reveal that SNHG14 is targeted by miR-2861 during the hMSCs differentiation to osteoblast.

According to previous reports, ALP is highly expressed in osteoblast, which is an important indicator for mature differentiation of osteoblast [19]. Osteoblast-specific genes *RUNX2*, *Osterix*, *ALP* and *OC* are also critical genes to indicate the existing of osteoblast [20, 21]. To investigate the effects of SNHG14 on hMSC osteoblast differentiation, we induced hMSCs differentiation to osteoblasts after transfection with shSNHG14 or shNC. The expression of SNHG14 was suppressed in cells transfected with shSNHG14. Suppression of SNHG14 markedly lowered osteoblastic differentiation, which was indicated by lower expression levels of the osteoblast-specific genes *RUNX2*, *Osterix*, *ALP*, and *OC*, decreased ALP activity, and in vitro matrix mineralization by Alizarin red staining in shSNHG14 transfected hMSCs compared with cells transfected with shNC. Similar to previous reports [15, 22], we also observed that silencing of SNHG14 could suppress hMSCs differentiation to osteoblasts.

A novel regulation role of Runx2/miR-3960/miR-2861 was demonstrated in mouse osteoblast differentiation [13]. MiR-2861 was found to promote osteoblast differentiation by increasing the expression of Runx2 [13]. To investigate the effects of miR-2861 on hMSC osteoblast differentiation, we induced hMSCs to differentiate to osteoblasts after transfection with miR-2861-mimic or miR-NC. Over-expression of miR-2861 greatly suppressed osteoblastic differentiation, which was indicated by lower expression levels of the osteoblast-specific genes *RUNX2*, *OSX*, *ALP*, and *OC, and* decreased ALP activity, and reduced in vitro matrix mineralization in miR-2861-mimic transfected hMSCs, compared to cells transfected with miR-NC. Different from the previous study [13], we noticed that miR-2861 suppressed osteoblast differentiation of hMSC. Moreover, we observed that the effects of SNHG14 on osteoblast differentiation of hMSC were attenuated by miR-2861.

## Conclusions

In conclusion, our data confirmed that the induction effects of SNHG14 on osteoblast differentiation of hMSCs were attenuated by miR-2861. SNHG14 could induce osteogenic differentiation of hMSC in vitro by targeting miR-2861.

## Supplementary information

**Additional file 1.**

## Data Availability

The analyzed data sets generated during the study are available from the corresponding author on reasonable request.

## Abbreviation

hMSCs: Human bone marrow mesenchymal stem cell

## References

[1] Aggarwal S, Pittenger MF (2005). Human mesenchymal stem cells modulate allogeneic immune cell responses. Blood.

[2] Sonoyama W, Liu Y, Fang D, Yamaza T, Seo B-M, Zhang C, Liu H, Gronthos S, Wang C-Y, Shi S (2006). Mesenchymal stem cell-mediated functional tooth regeneration in swine. PLoS One.

[3] Nuttelman CR, Tripodi MC, Anseth KS (2006). Dexamethasone-functionalized gels induce osteogenic differentiation of encapsulated hMSCs. J Biomed Mater Res Part A.

[4] Dawson E, Mapili G, Erickson K, Taqvi S, Roy K (2008). Biomaterials for stem cell differentiation. Adv Drug Deliv Rev.

[5] Nguyen MK, Jeon O, Krebs MD, Schapira D, Alsberg E (2014). Sustained localized presentation of RNA interfering molecules from in situ forming hydrogels to guide stem cell osteogenic differentiation. Biomaterials.

[6] Eskildsen T, Taipaleenmäki H, Stenvang J, Abdallah BM, Ditzel N, Nossent AY, Bak M, Kauppinen S, Kassem M (2011). MicroRNA-138 regulates osteogenic differentiation of human stromal (mesenchymal) stem cells in vivo. Proc Natl Acad Sci.

[7] Yang G, Lu X, Yuan L (2014). LncRNA: a link between RNA and cancer. Biochim Biophys Acta.

[8] Voorhoeve PM, Le Sage C, Schrier M, Gillis AJ, Stoop H, Nagel R, Liu Y-P, Van Duijse J, Drost J, Griekspoor A (2006). A genetic screen implicates miRNA-372 and miRNA-373 as oncogenes in testicular germ cell tumors. Cell.

[9] Zhu L, Xu P-C (2013). Downregulated LncRNA-ANCR promotes osteoblast differentiation by targeting EZH2 and regulating Runx2 expression. Biochem Biophys Res Commun.

[10] Liao J, Yu X, Hu X, Fan J, Wang J, Zhang Z, Zhao C, Zeng Z, Shu Y, Zhang R (2017). lncRNA H19 mediates BMP9-induced osteogenic differentiation of mesenchymal stem cells (MSCs) through notch signaling. Oncotarget.

[11] Qi X, Shao M, Sun H, Shen Y, Meng D, Huo W (2017). Long non-coding RNA SNHG14 promotes microglia activation by regulating miR-145-5p/PLA2G4A in cerebral infarction. Neuroscience.

[12] Hamam D, Ali D, Vishnubalaji R, Hamam R, Al-Nbaheen M, Chen L, Kassem M, Aldahmash A, Alajez N (2014). microRNA-320/RUNX2 axis regulates adipocytic differentiation of human mesenchymal (skeletal) stem cells. Cell Death Dis.

[13] Hu R, Liu W, Li H, Yang L, Chen C, Xia Z-Y, Guo L-J, Xie H, Zhou H-D, Wu X-P (2011). A Runx2/miR-3960/miR-2861 regulatory feedback loop during mouse osteoblast differentiation. J Biol Chem.

[14] Zuo C, Wang Z, Lu H, Dai Z, Liu X, Cui L (2013). Expression profiling of lncRNAs in C3H10T1/2 mesenchymal stem cells undergoing early osteoblast differentiation. Mol Med Rep.

[15] Huang Y, Zheng Y, Jia L, Li W (2015). Long noncoding RNA H 19 promotes osteoblast differentiation via TGF-β1/S mad3/HDAC signaling pathway by deriving mi R-675. Stem Cells.

[16] Tye CE, Gordon JA, Martin-Buley LA, Stein JL, Lian JB, Stein GS (2015). Could lncRNAs be the missing links in control of mesenchymal stem cell differentiation?. J Cell Physiol.

[17] Schoolmeesters A, Eklund T, Leake D, Vermeulen A, Smith Q, Aldred SF, Fedorov Y (2009). Functional profiling reveals critical role for miRNA in differentiation of human mesenchymal stem cells. PLoS One.

[18] M Kumar M, Goyal R (2017). LncRNA as a therapeutic target for angiogenesis. Curr Top Med Chem.

[19] Mizuno M, Kuboki Y (2001). Osteoblast-related gene expression of bone marrow cells during the osteoblastic differentiation induced by type I collagen. J Biochem.

[20] Jang W-G, Kim E-J, Kim D-K, Ryoo H-M, Lee K-B, Kim S-H, Choi H-S, Koh J-T (2012). BMP2 protein regulates osteocalcin expression via Runx2-mediated Atf6 gene transcription. J Biol Chem.

[21] Salingcarnboriboon R, Tsuji K, Komori T, Nakashima K, Ezura Y, Noda M (2006). Runx2 is a target of mechanical unloading to alter osteoblastic activity and bone formation in vivo. Endocrinology.

[22] Lu Y-F, Liu Y, Fu W-M, Xu J, Wang B, Sun Y-X, Wu T-Y, Xu L-L, Chan K-M, Zhang J-F (2016). Long noncoding RNA H19 accelerates tenogenic differentiation and promotes tendon healing through targeting miR-29b-3p and activating TGF-β1 signaling. FASEB J.

