# Supplementary figures and images for: SNHG14 induces osteogenic differentiation of human stromal (mesenchymal) stem cells in vitro by downregulating miR-2861
Source: BMC Musculoskelet Disord. 2020 Aug 8;21:525. doi: 10.1186/s12891-020-03506-9 (PMC7415173; doi:10.1186/s12891-020-03506-9)

Figure 6C


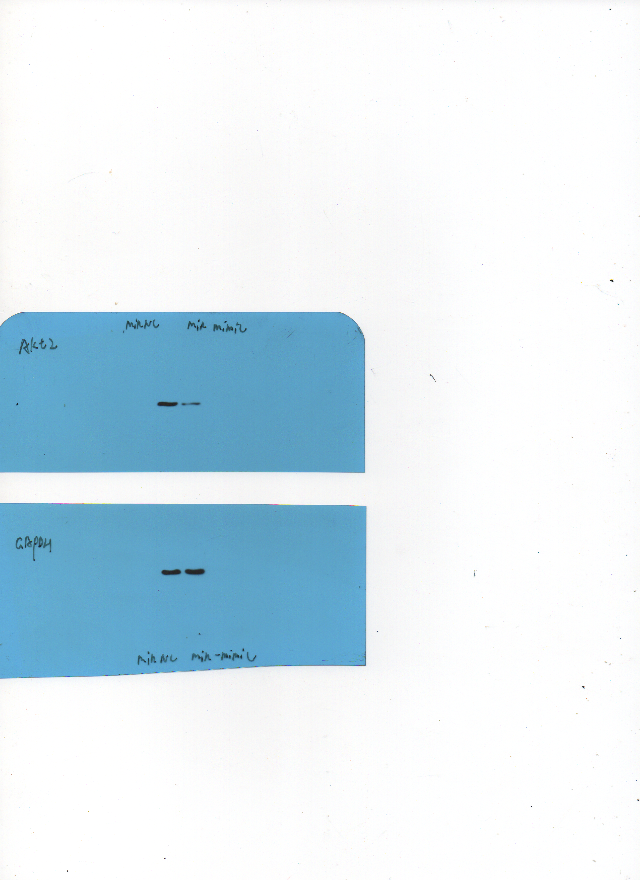


Figure 6D


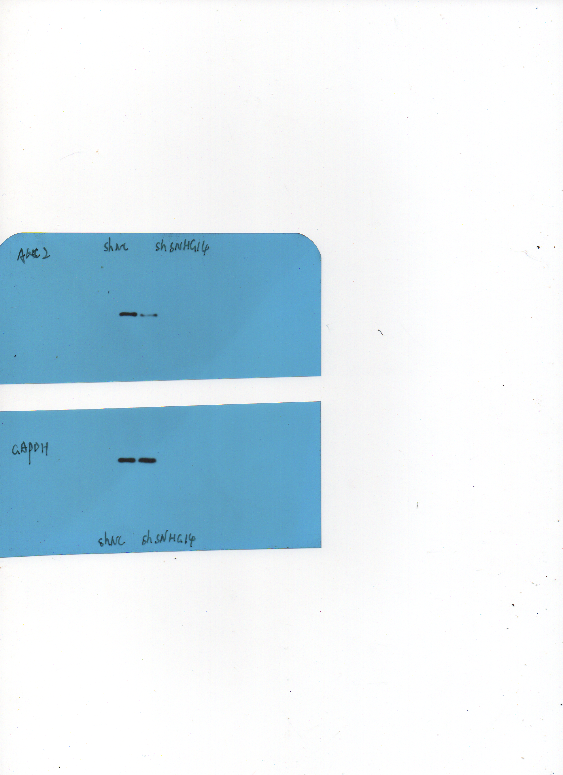

Supplement: Supplementary file 1 — Additional file 1. [file 12891_2020_3506_MOESM1_ESM.docx]
